# Malaria-Associated Spontaneous Splenic Rupture With Coagulopathy and Hemodynamic Compromise: A Case Report

**DOI:** 10.7759/cureus.77605

**Published:** 2025-01-17

**Authors:** Sarah M Mohammad, Esraa O Alsayed, Amal N Alharbi

**Affiliations:** 1 Medicine, Taibah University, Medina, SAU; 2 Radiology, King Fahad General Hospital, Medina, SAU

**Keywords:** conservative, malaria, plasmodium vivax, splenic rupture, spontaneous

## Abstract

Spontaneous splenic rupture is a rare, life-threatening condition that occurs without trauma, often presenting diagnostic challenges due to nonspecific symptoms. The risk of spontaneous splenic rupture increases significantly with underlying pathological conditions, including *Plasmodium vivax* malaria, where rapid splenic enlargement and altered red blood cell surface characteristics likely contribute to the increased risk of rupture. Here, we describe the case of a 23-year-old male patient who presented with severe abdominal pain and hypotension. Examination revealed jaundice, left upper abdominal tenderness, and hypotension. Laboratory findings showed thrombocytopenia and coagulopathy, while CT imaging revealed hemoperitoneum and splenic rupture. *P. vivax* malaria was confirmed, and the patient recovered fully with clinical monitoring and conservative management, including blood transfusions, antibiotics, and antimalarials, without the need for surgery. Splenic rupture in *P. vivax* malaria is believed to result from rapid splenic hyperplasia and vascular congestion. The role of early imaging, particularly CT, is crucial in confirming the diagnosis. Conservative management in stable patients appears effective, avoiding the risks associated with splenectomy. In conclusion, spontaneous splenic rupture, though rare, requires high clinical suspicion in *P. vivax* malaria-endemic regions. It should be considered in patients with sudden abdominal pain and instability, even in the absence of trauma. Further research into its pathophysiology and risk factors is essential for earlier diagnosis and improved management.

## Introduction

Spontaneous splenic rupture, a severe condition most frequently linked to abdominal or chest trauma, can rarely occur without prior trauma [1]. First documented in the 19th century, it has been reported by various researchers in subsequent years. Non-traumatic splenic rupture is categorized into six etiological groups: infectious, neoplastic, inflammatory, congenital or structural, iatrogenic, and idiopathic [2]. The diagnosis of spontaneous splenic rupture poses significant challenges. However, the criteria established by Orloff and Peskin, which require fulfillment of four specific conditions, can aid in the diagnostic process. These criteria include (1) a comprehensive medical history that reveals no antecedent trauma, (2) the absence of pathological conditions in other organs that might contribute to the rupture, (3) no perisplenic adhesions or scarring suggestive of prior trauma or rupture and (4) a normal appearance of the spleen upon both gross and histological examinations [3]. Spontaneous splenic rupture management depends on the splenic injury's severity. Mild cases may be treated conservatively with fluid administration and close monitoring, while more severe cases may require interventions such as splenic artery embolization, splenic salvage, or splenectomy if conservative measures do not achieve hemodynamic stability [3].

Recent reports have highlighted spontaneous splenic rupture as a complication associated with various conditions, including malaria, aortic valve replacement for bacterial endocarditis, factor VIII deficiency, and autologous transplantation for primary systemic amyloidosis [4]. Among these, malaria stands out as a critical cause of splenic rupture due to its pathophysiology. Malaria, caused by *Plasmodium *parasites and transmitted by mosquitoes, can lead to severe complications such as shock, respiratory distress, severe anemia, seizures, and inflammation of intra-abdominal organs. Among these complications, spontaneous splenic rupture can occur in up to 2% of malaria cases, though it remains rare [5]. This association underscores the importance of considering spontaneous splenic rupture as a potential complication in patients with malaria. We report a case of a patient who presented to the emergency room complaining of abdominal pain and was later diagnosed with malaria-related splenic rupture.

## Case presentation

A 23-year-old Indian man, with no significant past medical history, presented to the emergency department with a three-day history of severe abdominal pain and fever. He arrived in Saudi Arabia eight months ago and had no other travel history. There were no changes in bowel habits or recurrent fever episodes. The patient reported no preceding trauma, allergies, previous hospital admissions, or surgeries. On physical examination, he appeared ill, drowsy, and jaundiced. His abdomen was distended, with generalized tenderness most pronounced in the left upper quadrant and right lower quadrant. There was no rebound tenderness. He was hypotensive, with a blood pressure reading of 82/72 mmHg.

Initial laboratory investigations revealed a venous blood gas (VBG) with a pH of 7.32, with pCO_2_ at 49 mmHg, HCO_3_ at 22 mmol/L, and an elevated lactate level at 4.4 mmol/L (Table 1). The complete blood count (CBC) showed a white blood cell (WBC) count of 20,000/mm³. Notably, the hemoglobin (Hb) level dropped from 13 g/dL to 10 g/dL within one hour, which, along with a platelet count of 19,000/mm³, raised concerns for acute hemorrhage and possible disseminated intravascular coagulation (DIC). Liver function tests showed normal aspartate aminotransferase (AST) and alanine aminotransferase (ALT) levels, but an elevated alkaline phosphatase (ALP) level of 157 U/L and a total bilirubin level of 33 µmol/L, with conjugated bilirubin at 7 µmol/L. Renal function tests were within normal limits. The coagulation profile was abnormal, with a prolonged prothrombin time (PT) of 23 seconds, a partial thromboplastin time (PTT) of 45 seconds, and an international normalized ratio (INR) of 1.76, further supporting the diagnosis of DIC.

**Table 1:** Patient's lab results WBC: white blood cell, Hb: hemoglobin, AST: aspartate aminotransferase, ALT: alanine aminotransferase, ALP: alkaline phosphatase, PT: prothrombin time, PTT: partial thromboplastin time, INR: international normalized ratio

Test	Result	Reference range
Venous blood gas (VBG)		
pH	7.32	7.35–7.45
pCO₂	49 mmHg	35–45 mmHg
HCO₃	22 mmol/L	22–28 mmol/L
Lactate	4.4 mmol/L	0.5–2.2 mmol/L
Complete blood count (CBC)		
WBC	20,000/mm³	4,000–11,000 cells/mm³
Hb	Dropped from 13 to 10 g/dL	13.5–17.5 g/dL
Platelets	19,000/mm³	150,000–450,000/µL
Renal function tests	Normal	-
Liver function tests		
AST-ALT	Normal	-
ALP	157 U/L	44–147 U/L
Total bilirubin	33 µmol/L	5–21 µmol/L
Conjugated bilirubin	7 µmol/L	0–7 µmol/L
Coagulation profile		
PT	23 seconds	11–13.5 seconds
PTT	45 seconds	25–35 seconds
INR	1.76	0.8–1.1

**Figure 1:**
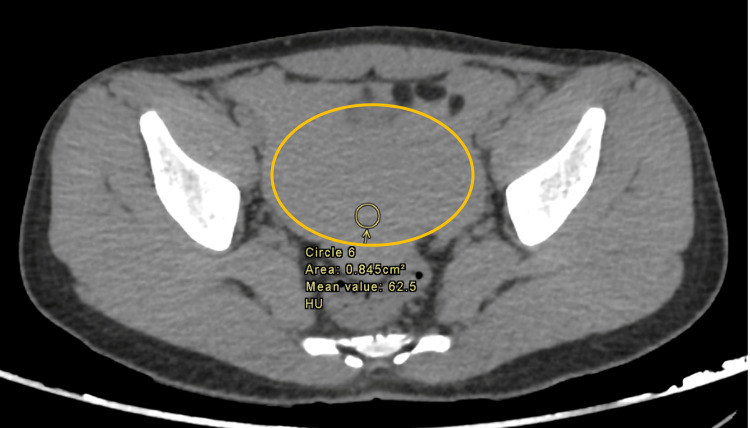
Abdominal CT scan (precontrast) demonstrating mild-moderate intra-abdominal hemoperitoneum (circle)

**Figure 2:**
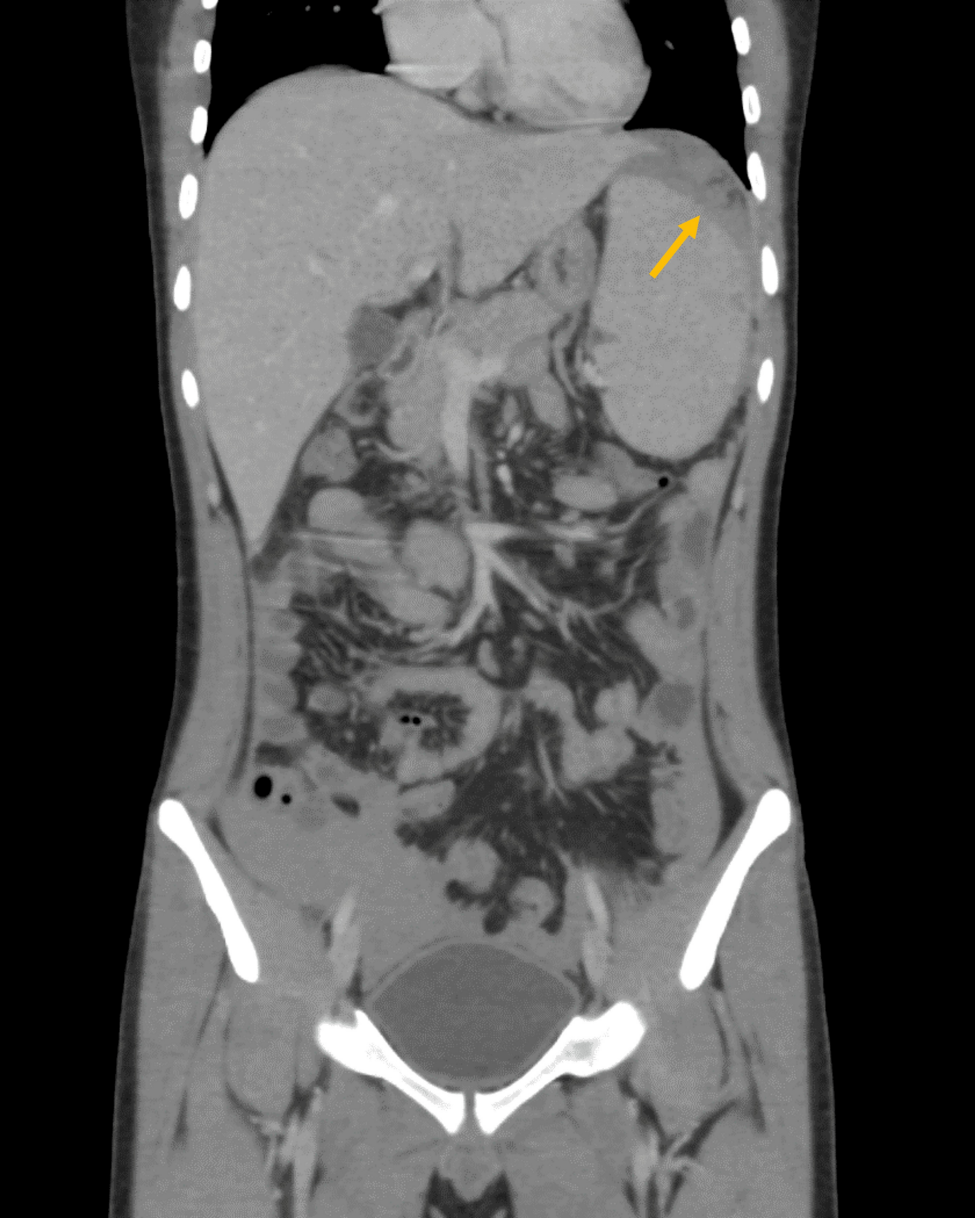
Abdominal CT scan (portal venous phase) with an IV contrast coronal cut demonstrating a mildly enlarged spleen with at least two small peripherally located splenic lacerations (arrow) measuring approximately up to 1 cm

**Figure 3:**
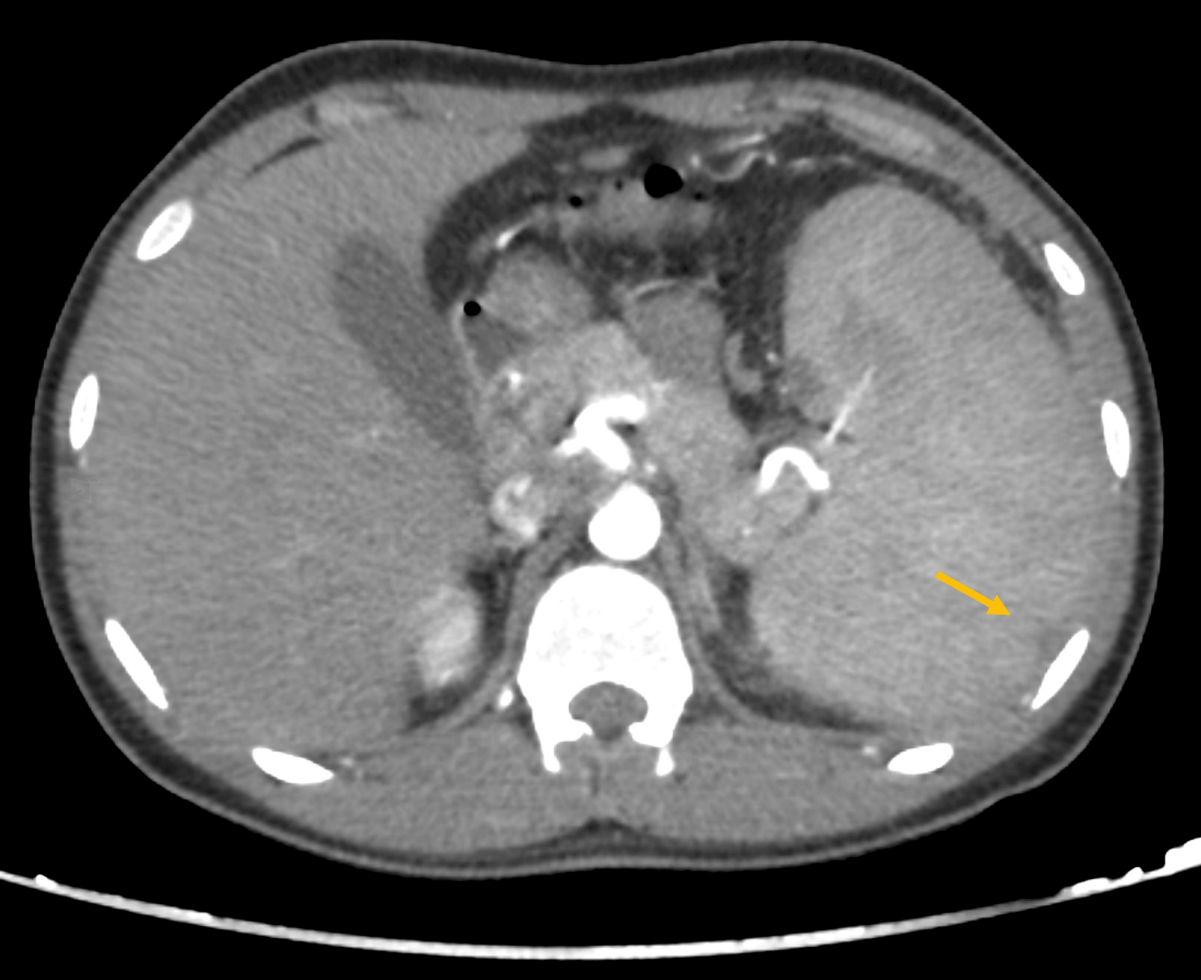
Abdominal CT scan (arterial phase) with an IV contrast axial cut demonstrating a mildly enlarged spleen with at least two small peripherally located splenic lacerations (arrow) measuring approximately up to 1 cm

Enhanced abdominal CT imaging revealed mild splenomegaly, with a calculated splenic ellipsoid volume of 535.5 mL, compared to the average normal spleen volume of 236.89 ± 77.58 mL [6]. There were signs of splenic rupture, with at least two peripheral splenic lacerations present, associated with mild to moderate intra-abdominal hemoperitoneum and no active arterial contrast extravasation. These findings are illustrated in Figures 1-3. Given the imaging findings and the lab results, a working diagnosis of splenic rupture with secondary peritonitis was considered, and the underlying infectious etiology was suspected.

The patient was closely monitored by the General Surgery team and underwent an infectious diseases evaluation. A rapid blood assay confirmed the presence of the *Plasmodium vivax* antigen, and the diagnosis was further validated by a peripheral blood smear, which identified the parasite. This workup indicated *P. vivax* malaria as a likely contributor to the splenic rupture and associated complications.

Initial management

The patient was given supportive care, including three units of packed red blood cells (PRBCs); nil per os (NPO, nothing by mouth) status was maintained, with intravenous fluids (D5 1/2 normal saline at 120 mL/h) and antibiotics (ceftriaxone 1 g IV twice daily, metronidazole 500 mg IV three times daily). For symptom relief, paracetamol 1 g IV three times daily was given for fever, and omeprazole 40 mg orally once daily was prescribed.

After initial treatment, the patient's hemoglobin improved to 12, and we decided against splenectomy. Although splenectomy is the usual treatment for splenic rupture caused by malaria, it comes with significant risks, especially infections. In areas with malaria, splenectomy can make future malaria infections more dangerous and requires long-term prevention. Splenectomy also increases the risk of infections such as pneumococcus, *Haemophilus influenzae*, and *Neisseria meningitidis* infections, which are common in tropical regions. Additionally, it may affect the immune response to diseases like schistosomiasis [7]. For these reasons, we chose to preserve the spleen, especially since the patient had exposure to tropical regions.

Antimalarial treatment

Upon confirmation of malaria, the patient was started on an antimalarial regimen based on the local malaria management guidelines in Saudi Arabia and the WHO recommendations. The treatment included artesunate (AS) 150 mg IV every 12 hours for two doses, followed by once-daily dosing, sulfadoxine/pyrimethamine (SP) 1500/75 mg orally as a single dose, and primaquine 0.5 mg/kg orally once daily for seven days. According to the local guidelines, the first-line treatment for malaria consists of AS combined with SP. Additionally, a single dose of primaquine (0.25 mg base/kg body weight, with a maximum dose of 15 mg) should be administered on the first day of treatment as a gametocytocidal agent to reduce transmission [8]. The patient's regimen adhered to these guidelines, with adjustments made to ensure proper dosing and duration of therapy.

Follow-up and outcome

After six days of treatment, repeat malaria screening results came negative, indicating a successful clearance of parasitemia. The patient exhibited significant clinical improvement, regaining consciousness, orientation, and hemodynamic stability. His abdomen was soft, non-tender, and less distended, reflecting the resolution of the acute abdominal findings. The patient was discharged in a stable condition, with instructions for follow-up care.

## Discussion

Spontaneous rupture of the spleen is a rare and potentially life-threatening condition that occurs without identifiable external trauma, distinguishing it from traumatic splenic rupture, which is typically linked to physical injury. Due to its rarity and the absence of clear precipitating factors, spontaneous splenic rupture presents unique diagnostic and therapeutic challenges, with an incidence estimated at less than 0.5% of all splenic rupture cases [9]. Conversely, pathological splenic rupture is often associated with underlying conditions, particularly infections such as malaria, where the splenic rupture represents a serious complication [9]. Malaria-related splenic rupture is primarily observed among individuals who have traveled to endemic regions; the median age is reported to be 31.5 years for affected patients, although cases span from 3 to 80 years of age. There is a notable male predominance with a sex ratio of 3.2:1, and travelers represent a higher risk group; however, splenic rupture can also occur in residents of malaria-endemic areas [10]. Among the *Plasmodium* species, *P. vivax* is most commonly associated with splenic rupture, likely due to its propensity to cause significant splenomegaly compared to *Plasmodium falciparum* [10]. A case of splenic rupture in *Plasmodium knowlesi* malaria infection was reported by Chang et al. [11]. In malaria-endemic regions, splenic enlargement is frequent, affecting 50%-80% of certain populations. Splenic enlargement generally becomes palpable within three to four days of symptom onset and can worsen if untreated, correlating with higher malaria prevalence and larger spleen sizes. While chronic malaria may contribute to significant splenic enlargement, spontaneous rupture typically occurs during acute infection episodes, especially during an initial attack [12]. Rapid hyperplasia, stretching of the splenic parenchyma and capsule, vascular occlusion, and micro-infarctions are believed to contribute to the risk of rupture. Notably, even minor activities that increase intra-abdominal pressure, such as sneezing or coughing, can precipitate rupture in a spleen compromised by malaria [12-14]. In addition, activation of the spleen's lymphatic tissue and congestion in its sinuses due to deformed red blood cells with altered surface characteristics may further increase the vulnerability to rupture [15].

Clinically, splenic rupture, whether spontaneous or pathological, often presents with nonspecific symptoms, including abdominal pain, tenderness, and possibly guarding or rigidity in the left upper quadrant. A characteristic symptom is Kehr’s sign, where pain in the left shoulder arises due to diaphragmatic irritation from perisplenic effusion [10]. In malaria cases, additional symptoms like splenomegaly, nausea, and fever may be present, depending on the timing of the rupture relative to the acute malarial episode. If undiagnosed, splenic rupture can progress rapidly to hypovolemic shock, with ensuing hypotension and anemia. Pathological splenic rupture in malaria is often underrecognized during life, leading to diagnosis primarily at autopsy, underscoring the need for heightened clinical vigilance in patients from endemic regions or those who have traveled recently [10]. An early diagnostic workup of suspected splenic rupture includes laboratory tests, though initial Hb levels may not reflect hemodilution changes. A baseline Hb level, packed cell volume (PCV), and cross-matching for transfusion support are essential in managing these patients [12]. Imaging modalities are central to the diagnostic process, with ultrasound being a preferred first-line tool due to its sensitivity (72%-78%) and high specificity (91%-100%), as well as its accessibility, portability, and minimal preparatory requirements [16]. In cases where greater detail is needed, CT of the abdomen provides critical information, identifying small subcapsular hematomas and enabling careful monitoring in conservatively managed cases [17]. Other diagnostic options include positron emission tomography (PET) and diagnostic peritoneal lavage [12]. The management of splenic rupture is guided by the patient’s hemodynamic stability. Hemodynamically stable patients can often be managed conservatively, with close imaging surveillance to monitor healing over a two- to three-week period. However, splenectomy becomes necessary for patients with ongoing shock or those who do not respond adequately to resuscitation. Due to the heightened risk of post-splenectomy infections, particularly in pediatric patients, clinicians generally aim to avoid splenectomy whenever feasible, especially in individuals at future risk of malaria exposure, as splenic preservation supports better long-term outcomes [14]. Splenectomy should be reserved for patients with severe rupture, continued or recurrent bleeding, or when rupture is secondary to neoplastic disease [9,12]. In recent cases, splenic artery embolization has been employed as an effective alternative to splenectomy, reducing perioperative complications, as demonstrated in a case from the Inha University Hospital, Incheon, Korea [18]. In the present case, the patient was closely monitored and received supportive care, resulting in significant clinical improvement that obviated the need for surgical intervention. He was discharged in a stable condition with instructions for follow-up.

## Conclusions

Spontaneous splenic rupture, though rare, poses a significant risk and requires high clinical suspicion, particularly in malaria-endemic regions with *Plasmodium vivax* prevalence. This case underscores the need to consider splenic rupture in patients with sudden abdominal pain and instability, even in the absence of trauma, as well as the importance of prompt antimalarial treatment when indicated. Further research into its unclear pathophysiology and risk factors is essential to facilitate earlier diagnosis and improve management for at-risk patients.
